# Dissection of hyperspectral reflectance to estimate nitrogen and chlorophyll contents in tea leaves based on machine learning algorithms

**DOI:** 10.1038/s41598-020-73745-2

**Published:** 2020-10-15

**Authors:** Hiroto Yamashita, Rei Sonobe, Yuhei Hirono, Akio Morita, Takashi Ikka

**Affiliations:** 1 Faculty of Agriculture, Shizuoka University, Shizuoka, Japan; 2 United Graduate School of Agricultural Science, Gifu University, Gifu, Japan; 3 Division of Tea Research, Institute of Fruit Tree and Tea Science, National Agriculture and Food Research Organization (NARO), Shimada, Japan

**Keywords:** Biological techniques, Computational biology and bioinformatics, Plant sciences

## Abstract

Nondestructive techniques for estimating nitrogen (N) status are essential tools for optimizing N fertilization input and reducing the environmental impact of agricultural N management, especially in green tea cultivation, which is notably problematic. Previously, hyperspectral indices for chlorophyll (Chl) estimation, namely a green peak and red edge in the visible region, have been identified and used for N estimation because leaf N content closely related to Chl content in green leaves. Herein, datasets of N and Chl contents, and visible and near-infrared hyperspectral reflectance, derived from green leaves under various N nutrient conditions and albino yellow leaves were obtained. A regression model was then constructed using several machine learning algorithms and preprocessing techniques. Machine learning algorithms achieved high-performance models for N and Chl content, ensuring an accuracy threshold of 1.4 or 2.0 based on the ratio of performance to deviation values. Data-based sensitivity analysis through integration of the green and yellow leaves datasets identified clear differences in reflectance to estimate N and Chl contents, especially at 1325–1575 nm, suggesting an N content-specific region. These findings will enable the nondestructive estimation of leaf N content in tea plants and contribute advanced indices for nondestructive tracking of N status in crops.

## Introduction

Nitrogen (N) is the most demanded element for photosynthetic function and growth in plants. N fertilization has become a major yield-enhancing technique in modern crop production^1–3^. However, excessive N fertilization is known to be a source of water and air pollution. Groundwater contamination by nitrate–N (NO_3_-N) from excess N fertilizer is a serious problem in many countries^4^. Furthermore, N fertilizers are important sources of nitrous oxide (N_2_O), which is involved in destruction of the atmospheric ozone layer^5,6^. Furthermore, excess N fertilizer application increases management costs, even in modern large-scale agricultural production. Therefore, optimizing the amount of N fertilization by tracking N status is necessary for crop nutrient status and to reduce the environmental impact of agricultural management.

N is a structural element of chlorophyll (Chl), affecting leaf greenness and Chl accumulation^7–9^. The proportion of leaf N allocated to the chloroplast is approximately 75%^10,11^. The leaf N content and Chl content in plant green leaf are positively correlated. This has been reported for numerous plant species, and nondestructive and rapid N status estimation has been conducted using Chl meters on most major crops, including rice (*Oryza sativa* L.)^12,13^, wheat *(Triticum aestivum* L.)^14^, maize (*Zea mays* L.)^15^, and others^16,17^. Nondestructive N status estimation in the leaf and canopy using hyperspectral sensing has also been applied to many plant species for nutritional diagnosis^18–25^. In most cases, the hyperspectral indices for Chl estimation, namely a green peak and red edge (500–800 nm) in the visible region, have been identified and used owing to their multicollinearity, with the leaf N content closely related to Chl content in plant green leaves, as mentioned above^12,22,25,26^. However, decreased Chl content can be caused by various factors, such as herbicide injury, that are not necessarily related to N deficiency^27^. Therefore, estimations of Chl and N content must be decoupled from remote sensing data to assess various stresses and pathogens^26^.

Machine learning techniques are powerful tools for estimating agricultural indices from hyperspectral remote sensing data^28^. Among the main advantages of machine learning algorithms is their ability to autonomously solve large nonlinear problems using datasets from multiple variables, and provide a powerful and flexible framework not only for data-driven decision making, but also for incorporating expert knowledge into the algorithms^29^. This methodology also shows potential for analyzing hyperspectral reflectance data with a large number of bands, working with not only variables such as derived spectral indices, but also all spectral information^30^. Previous spectral indices have depended on a small number of available spectral bands and, therefore, do not use all information conveyed by the spectral trace^29^. Machine learning techniques can assess the features that are more informative for high-accuracy prediction modelling^29,31^.

Tea plants (*Camellia sinensis* L.) are mainly cultivated in Asia for the production of green, oolong, and black teas, which are among the most popular beverages worldwide. Tea is a leaf-harvested crop, and N is the most important nutrient for improving the yield and quality, such as free amino acid contents, of tea leaves^32^. Therefore, to meet these criteria, tea fields, especially in Japan, tend to receive higher rates of N fertilization, such as with ammonium sulfate, than other crops, sometimes exceeding 1000 kg N ha^−1^ year^−1^^33^. Heavy N fertilization in tea fields often causes problems such as increased NO_3_-N levels in surrounding water systems^34^ and high N_2_O emission levels^32,33,35,36^. N_2_O emission rates in tea fields are much higher than those in other upland fields and paddy fields^33,37^. Reducing NO_3_-N leaching and N_2_O emissions from tea fields would be a significant step towards decreasing the environmental impact of agricultural N management. In addition, tea plants with characteristic leaf colors, such as yellow (or white)^38–42^ and purple^43^, have been studied extensively. Mutant (bud-sport) branches with albino yellow leaves due to a lack of chlorophyll are often found in tea gardens, with albino-induced tea leaves generally containing higher amino acid contents than conventional green tea leaves^38–42^. Therefore, the possibility that albino tea leaves might have an N status that does not reflect the Chl status was considered.

This study mainly aimed to assess differences in hyperspectral reflectance to estimate N and Chl contents and enable nondestructive estimation of leaf N contents in tea plants, which require large amounts of N nutrition. Initially, the dataset of N and Chl contents and visible and near-infrared hyperspectral reflectance with variations derived from green leaves with various N nutrient conditions and albino yellow leaves was obtained. A regression model was then constructed using several machine learning algorithms and preprocessing techniques. Data-based sensitivity analysis based on high-performance models using integrating datasets from green and albino yellow leaves identified clear differences in reflectance to estimate N and Chl contents.

## Results

### Data distribution of nitrogen and chlorophyll contents

**Figure 1:**
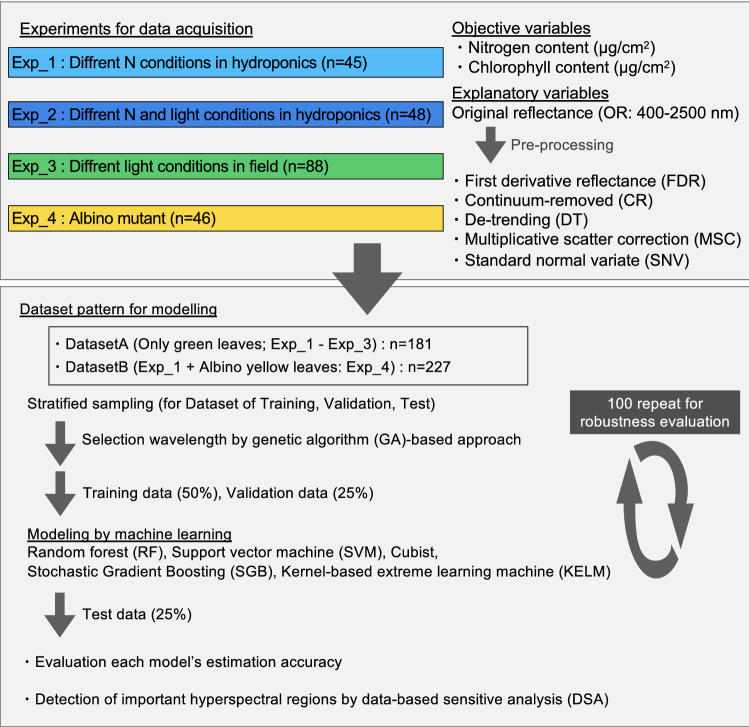
Experiment and modelling designs in this study.

**Figure 2:**
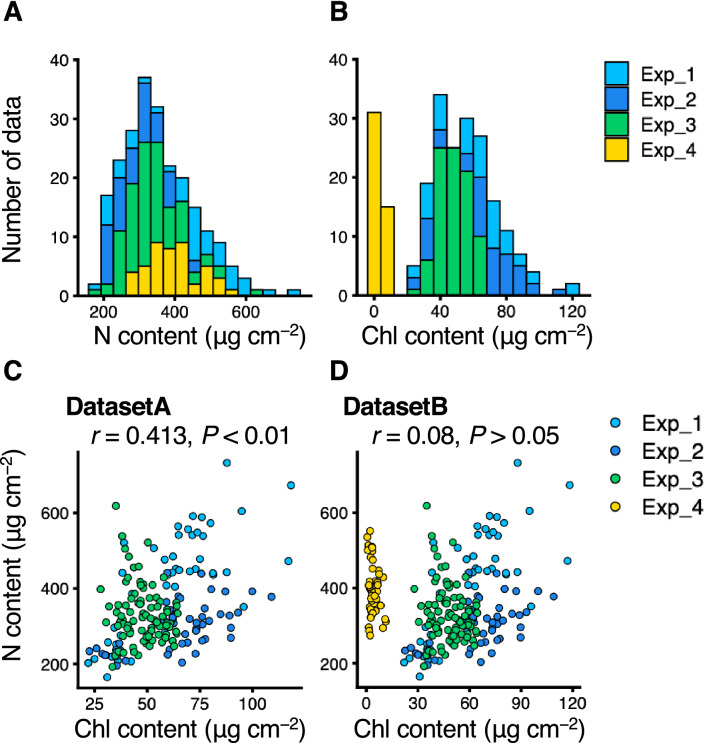
Data distributions of nitrogen (N) and chlorophyll (Chl) contents in tea leaves. Histograms of (**A**) N and (**B**) Chl contents in all experiments. Correlation plots of N and Chl contents in (**C**) DatasetA and (**D**) DatasetB. Figures were visualized by using the R package “ggplot2” ver. 3.3.2. Statistical results for correlation were shown on the figure.

To obtain the dataset of N and Chl contents with variations, green leaves (GL) with various N nutrient conditions from hydroponic and shading tests (Exp. 1 to Exp. 3) and albino yellow leaves (YL; Exp. 4) were tested (Fig. 1). In all experiments, the N and Chl contents were in the range of 164.8–732.5 and 0.61–118.3 mg cm^−2^, respectively (Figs. 2A,B). The dataset for subsequent modelling was divided into DatasetA (n = 181), comprising only the GL data (Exp. 1 to Exp. 3), and DatasetB (n = 227), comprising the GL and YL data (Exp. 1 to Exp. 4) (Fig. 1). A significant positive correlation was observed between N and Chl content in DatasetA, but not in DatasetB (Fig. 2C,D).

### Regression model performance

**Figure 3:**
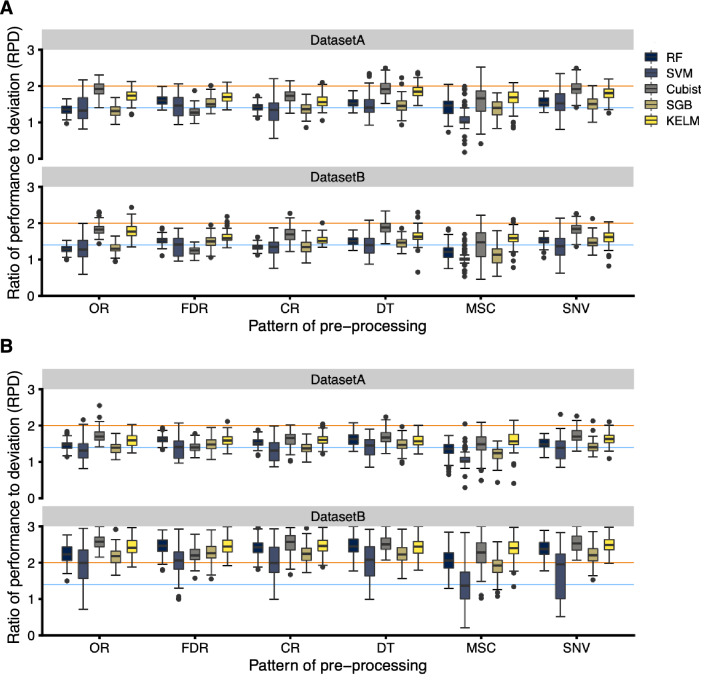
Model performance and robustness for each pre-processing of reflectance. Modelling with (**A**) nitrogen and (**B**) chlorophyll contents as the objective variables was performed using the explanatory variables of DatasetA and DatasetB. The ratio of performance to deviation (RPD) was applied to evaluate the accuracy of each model. A stratified sampling approach for modelling was repeated 100 times to obtain robust results. Figures are plots of the RPD values in each repeat. Orange and blue lines indicate RPD values of 1.4 and 2.0, respectively, as accuracy thresholds. Figures were visualized by using the R package “ggplot2” ver. 3.3.2.

Original reflectance (OR) data at 1-nm steps across the entire wavelength domain from 400 to 2500 nm was obtained from the leaf samples. Five preprocessing methods, namely first derivative reflectance (FDR), continuum-removed (CR), standard normal variate (SNV), multiplicative scatter correction (MSC), and de-trending (DT) (Supplementary Fig. S1), were applied to the OR data to compare regression model performance. The following five regression methods were performed: Random Forest (RF), Support Vector Machine (SVM), Cubist, Stochastic Gradient Boosting (SGB), and Kernel-based Extreme Learning Machine (KELM). Model performance was evaluated using the ratio of performance to deviation (RPD) values and robustness over 100 repetitions. For N content modelling, Cubist and KELM models indicated high performance and robustness when OR, DT, and SNV were applied as hyperspectral data both in DatasetA and DatasetB, as most RPD values over 100 repetitions were greater than 1.4, representing a fairly acceptable prediction level (Fig. 3A). For Chl content modelling, the same pattern of results as above was observed (Fig. 3B), and the model performance of DatasetB was higher than that of DatasetA, with most RPD values over 100 repetitions being above 2.0, representing an accurate prediction level (Fig. 3B). These results were also supported by the coefficient of determination (R^2^) and root mean square error (RMSE) values as model performance indices (Supplementary Figs. S2 and S3).

### Detection of important hyperparameters by data-based sensitivity analysis

**Figure 4:**
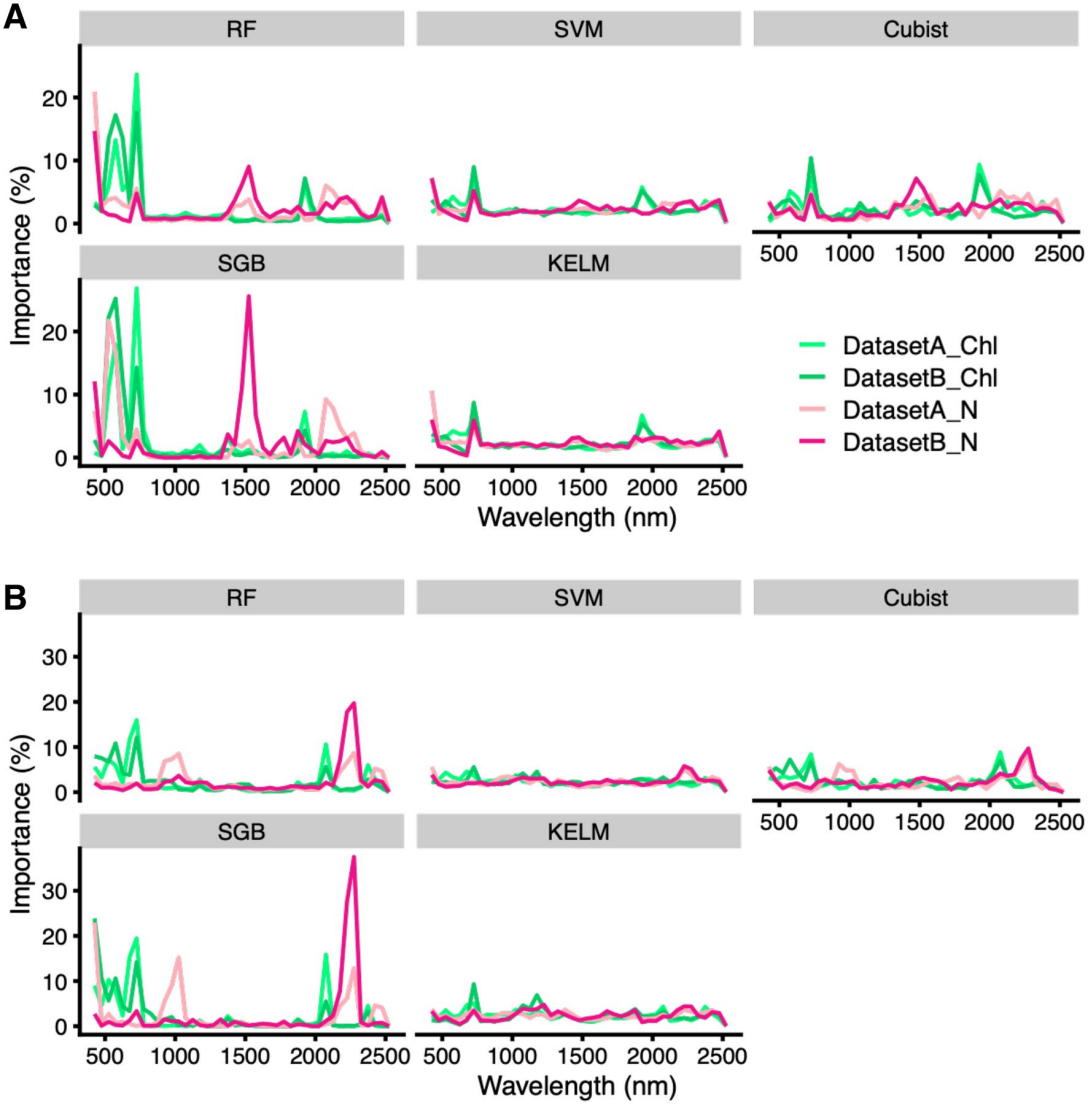
Detection of important hyperspectral parameter regions as model variables by data-based sensitive analysis (DSA). DSA results when (**A**) original reflectance or (**B**) de-trending (DT) were visualized as the explanatory variables in each model. Figures were visualized by using the R package “ggplot2” ver. 3.3.2.

Data-based sensitivity analysis (DSA) was performed to detect important hyperspectral parameters in models to estimate N and Chl contents. The results of DSA in models applying OR hyperspectral parameters are shown in Fig. 4A. For Chl content, both DatasetA and DatasetB showed peaks of importance at 525–725 and 1875–1925 nm in all models (Fig. 4A). For N content, peaks of importance were observed at 675–725 and 1325–1575 nm, with the latter peak enhanced in DatasetB compared with DatasetA, especially for RF, Cubist, and SGB (Fig. 4A). The DSA results of models applying DT hyperspectral parameters are shown in Fig. 4B. For Chl content, both DatasetA and DatasetB showed peaks of importance at 475–725 nm in all models, and 1975–2075 nm for RF, Cubist, and SGB (Fig. 4B). For N content, both DatasetA and DatasetB showed peaks of importance at 2125–2275 nm, especially for RF, Cubist, and SGB (Fig. 4B).

**Figure 5:**
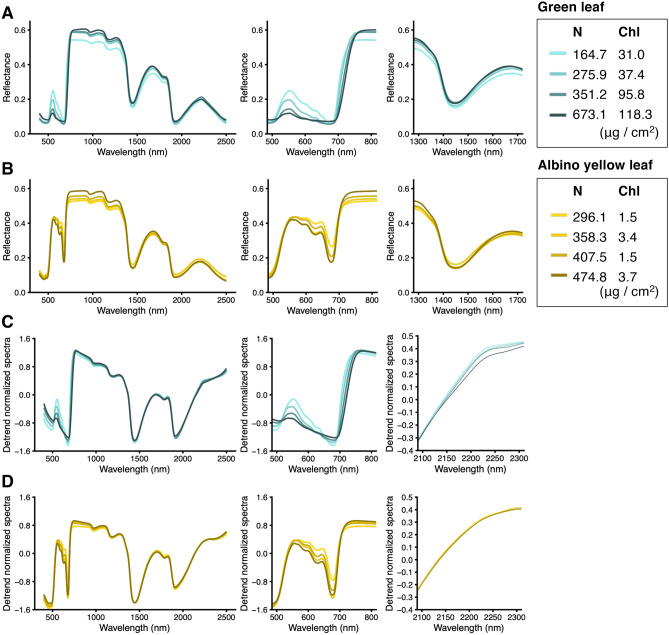
Typical original reflectance and de-trending spectra of green and albino yellow leaves with different nitrogen (N) statuses. Reflectance spectra of (**A**) green and (**B**) albino yellow leaves. De-trend normalized spectra of (**C**) green and (**D**) albino yellow leaves. N and chlorophyll contents in each spectrum are shown with different colors to the right of the figure. Each figure consists of all measured wavelengths (left), green peak and red edge regions (500–800 nm; middle), and important ranges identified by DSA (right). Figures were visualized by using the R package “ggplot2” ver. 3.3.2.

Typical OR and DT spectra, and their important regions based on DSA of GL and YL with different N statuses, are shown in Fig. 5. The OR spectra of GL showed a clear response to N and Chl contents at 500–800 nm in the green peak and red edge region (Fig. 5A). The OR spectra of YL showed high reflectance at around 550 nm in the green peak region, independent of N status (Fig. 5B). A shift dependent on N status toward shorter wavelengths in the 700–780 nm region (blue shift) was observed in GL, but not YL (Fig. 5A,B). In the OR spectra of GL and YL at 1300–1700 nm, a clear response to N content was observed (Fig. 5A,B). Furthermore, DT spectra of GL and YL showed a clear response to N and Chl contents at 500–800 nm in the green peak and red edge region (Fig. 5C,D). At 2100–2300 nm in the DT spectra of GL and YL, a clear response to N content was observed, especially for GL (Fig. 5C,D).

## Discussion

N fertilization, which is directly related to yield and quality, is indispensable in modern agriculture to achieve stable food production^1–3,32^. However, improving the efficiency of N nutrition is necessary for both agricultural management and global environmental conservation^4,6,33^. In green tea cultivation, these environmental impacts in agricultural N management have been notably problematic^33–35^. Therefore, the impact of N management can be maximized in tea cultivation. Nondestructive estimation techniques, such as hyperspectral reflectance, are effective tools for tracking crop status^18–25^. In previous studies, hyperspectral techniques have been used to estimate N status, such as for Chl in leaves^12,22,25^. Therefore, this study aimed to determine differences in hyperspectral reflectance to accurately estimate N and Chl contents in GL and YL using machine learning algorithms, and enable the nondestructive estimation of leaf N content in tea plants.

Datasets of N and Chl contents were obtained from GL under various N nutrient conditions in hydroponic and shading tests (Exp. 1 to Exp. 3) and YL (Exp. 4) (Fig. 2A,B), and DatasetA (n = 181; only GL) and DatasetB (n = 227; GL and YL) were constructed for subsequent modelling (Fig. 1). A reflectance in YL clearly showed a different appearance to that in GL (Fig. 5). The reflectance of the green peak region in YL was high and broad, merging together with the start point of the red edge region (Fig. 5B), as a characteristic of albino leaves reported by Baldini et al. (1997)^44^. In DatasetB, the positive correlation between N and Chl contents was not observed (Fig. 2D). This result suggested that modelling using DatasetB did not suffer from multicollinearity between the N and Chl contents, allowing the selection of explanatory variables specific to each.

In our modelling, machine learning methods, especially Cubist and KELM, with OR data showed high-performance and robustness both for N and Chl contents, ensuring accuracy thresholds based on the RPD values (Fig. 3). Cubist can generate so-called committee models that consist of a set of consecutive rule-based models to correct the predictions of previous member models^44^. Furthermore, Cubist is computationally efficient^44^ and well-suited to big data analytics^45^. Cubist has shown potential as an efficient model method for various agricultural targets using reflectance data, such as soil physical properties^46^, vegetation stress^47^, leaf area index^48^, and crop yields^49^. Although KELM and SVM are both kernel-based algorithms, SVM was clearly inferior to KELM in this modelling. Furthermore, the variance of the kernel function parameters of KELM was apparently smaller than that of SVM (Supplementary Table S1). Generally, the ranges of the kernel bandwidth of SVM (s) were wider than those of the kernel parameter of KELM (Kp), implying that the Bayesian optimization sometimes resulted in local solutions for optimizing SVM hyperparameters. Indeed, quite low RPD values (less than 1.0) for SVM were confirmed using all preprocessing techniques (Fig. 3). The incorrect selection of hyperparameters related to kernel function has been reported to reduce the estimation accuracy^50^, and local solutions of the Bayesian optimization led to worse estimation accuracies for SVM. Furthermore, KELM has fewer optimization constraints^51^, which is advantageous in regression applications^52^. The results of this and previous studies indicate that the Cubist and KELM methods are suitable for modelling using reflectance data in plants. Some preprocessing techniques, especially DT, also showed similar results to those using OR data. Barnes et al. (1989)^53^ reported that DT accounted for the variation in baseline shift and curvilinearity from the reflectance spectra of powdered or densely packed samples using second-degree polynomial regression. DT was an effective preprocessing technique for estimating the N and Chl contents, and the potential of DT for targeting plants was also confirmed in this study. Comparing DatasetA and DatasetB, the model performance for N content was similar (Fig. 3A). However, for Chl content, the model performance of DatasetB was higher than that of DatasetA, with most RPD values above 2.0 for 100 repetitions, representing an accurate prediction level (Fig. 3B). These results suggested that the inclusion of YL, which had the lowest Chl content in the dataset, functioned well at estimating the Chl content, but not the N content. Furthermore, these results indicated that the approach using hyperspectral reflectance and machine learning algorithm allowed not only the Chl content, as previously reported by Sonobe et al. (2020)^54^, but also the N content to be estimated nondestructively in tea leaves. For green tea production, especially in Japan, N fertilizer is mainly applied in the autumn, winter, and at the bud-opening stage before the first crops in spring, to improve the quality^55^. This is because N absorbed into the source organs, such as mature leaves, contributes to new shoots for the first crops^55^. Therefore, modelling was also conducted to estimate the N content in mature leaves, using only GL from this experiment design (Supplementary Fig. S4). This modelling approach provided the N content of mature leaves with high performance and robustness (Supplementary Figs. S5 and S6), but was limited by the possibility of multicollinearity with the Chl content (Supplementary Fig. S4C).

The important hyperspectral parameters in models to estimate N and Chl content were detected using DSA. For the Chl content in both DatasetA and DatasetB, peaks of importance were observed at 525–725 and 1875–1925 nm in all models (Fig. 4A). Chlorophyll absorbs energy strongly in the ultraviolet (200–400 nm), blue (400–500 nm), and red (650–690 nm) regions, and shows weak reflectance and transmittance^56^. Therefore, most indices for Chl content in plant leaves and canopies were selected wavelengths, as a few narrow bands, ranging from 400 to 860 nm^57–59^ or the red edge (680–750 nm)^60,61^. Previously, our analysis of Chl content in tea leaves also indicated that the values of highest importance were confirmed at 700–750 nm (the red edge region) when OR and FDR were used^54^. For N content, the peaks of importance were observed at 675–725 and 1325–1575 nm, with the latter peak in the near-infrared region enhanced in DatasetB compared with DatasetA, especially for RF, Cubist, and SGB (Fig. 4A). These results suggested that this near-infrared reflectance region, at 1325–1575 nm, was N content-specific, independent of the Chl content. Previous studies have shown that plant water status affects shortwave-infrared and near-infrared domains, showing that these domains are sensitive to water absorption^62–65^. No correlation was observed between the leaf water and N contents in the dataset of this study (Supplementary Fig. S7), which supported that the near-infrared reflectance region of 1325–1575 nm detected in this analysis was an N content-specific region. Furthermore, DSA with some preprocessing techniques also detected the different hyperspectral regions to estimate N and Chl content (Supplementary Fig. S8). Therefore, preprocessing techniques might be a tool for the specific estimation of N and Chl contents using hyperspectral data.

The results of this study suggest that hyperspectral reflectance data and machine learning techniques show good potential for estimating leaf N and Chl contents in tea plants. Remote sensing techniques using unmanned aerial vehicles will also enable the high-throughput estimation of N and Chl statuses in canopy-scale tea gardens. Furthermore, the inclusion of YL, which had the lowest Chl content in the dataset, contributed to the detection of N-content-specific hyperspectral regions, independent of Chl content, in the near-infrared reflectance region. These findings will contribute advanced indices for the nondestructive tracking of crop N status. In the future, these techniques could be applied to improve irregularities in fertilizer and the real-time diagnostics of physiological status changes in large farms.

## Methods

### Plant materials for datasets

To obtain the dataset of N and Chl contents with variations, the following four experiments (Exp. 1 to Exp. 4) were conducted.

Exp. 1 and Exp. 2 were conducted using hydroponic nutrient tests. The hydroponic culture of tea plants was conducted under sunlight conditions in the greenhouse at Shizuoka University (Shizuoka, Japan) using a slight modification of the method described by Konishi et al. (1985)^66^. One-year-old rooted tea cuttings of cv. ‘Yabukita’, a leading Japanese green tea cultivar, were transplanted with three individuals in Wagner pots (1/5000 a) containing tap water (3 L) adjusted to pH 4.2 using H_2_SO_4_, and continuously aerated. After 1 week, standard nutrient solutions containing N 40 mg L^−1^ (1 × N), 10 mg L^−1^ NO_3_-N, and 30 mg L^−1^ NH_4_-N at pH 4.2^66^ were supplied stepwise for 1 week each at 1/5, 1/2, and 1/1 strength to adapt the plants to the hydroponic system. Subsequently, the following experiments were performed.

In Exp. 1, plants were transferred to nutrient solutions adjusted to the following six N levels: –N, 0.01 × N, 0.1 × N, 1 × N, 2 × N, 4 × N. Each experiment was conducted using three to five biological replicates. Solutions were renewed every week. After approximately 6 months, new leaves, the second leaf from bottom in four-leaf-stage new shoots, and mature leaves were plucked from two to four spots in one replicate, and then leaf reflectance was measured. After measuring reflectance, the leaves were scanned at 300 dpi (CanonScan LiDE 210 JP scanner, Canon Inc, Tokyo, Japan) to calculate the leaf area, weighed in fresh condition, and then reweighed in dry condition after freeze-drying. Dry samples were grounded into a fine powder and stored at room temperature in a desiccator prior to nitrogen and chlorophyll measurements.

In Exp. 2, when new buds were developed, plants were transferred to nutrient solutions adjusted to the following four N levels: –N, 0.1 × N, 1 × N, 4 × N. The plants at each N level were grown under normal (control) and low-light (shading) conditions. Low-light conditions were set up with 85% coverage by synthetic black cloth (85P, Dio Chemicals, Tokyo, Japan). Each experiment was conducted using three biological replicates. The solutions were renewed every week. After 23 days, new leaves, the second leaf from bottom in four-leaf-stage new shoots, and mature leaves were plucked from one to two spots in one replicate, respectively, and measured as mentioned above.

In Exp. 3, new shoots were plucked from mature tea ridges of cv. Yabukita at Shizuoka University (Shizuoka, Japan) under open and low-light conditions with 85% shading. After developing the two leaves of almost new shoots in the first crop season (around the end of April), shaded cultivation was conducted. These tea ridges were shaded by 85% coverage with black cloth (85P, Dio Chemicals, Tokyo, Japan) for 20 days of the first crop season of spring in Japan. Shading materials directly covered the tea canopies, as described by Sano et al. (2018)^67^. After shade cultivation, new shoots and mature leaves were plucked from approximately 15 spots in open and shaded tea ridges, and measured as described above. New leaves were then plucked from the third, fourth, and fifth leaves in five-leaf-stage new shoots. Tea ridges were managed by conventional methods in Japan and cultivated under the same soil and environment conditions. Nitrogen fertilizer was applied at 400 kg-N ha^−1^ year^−1^.

In Exp. 4, new shoots were plucked from 7-year-old rooted tea cuttings of cv. ‘Koganemidori’, previously known as “Morokozawa”^38^, which had been bred from the natural albino mutant, and hydroponically cultivated under sunlight condition in the greenhouse at Shizuoka University (Shizuoka, Japan), as described above. This albino cultivar “Koganemidori” has a yellow color leaf and very low chlorophyll content (Supplementary Fig. S9)^38^. In the first crop season, new shoots were plucked from approximately 20 spots in one replicate and measured as described above. New leaves were then plucked from the second, third, and fourth leaves in four-leaf-stage new shoots.

### Reflectance measurements

An ASD FieldSpec4 unit (Analytical Spectral Devices, USA) was used to obtain reflectance data from leaf clippings (*φ*, 10 mm). This device contained three detectors, namely, visible and near-infrared (VNIR), short wave infrared (SWIR) 1, and SWIR 2. The splice correction function of ViewSpec Pro Software (Analytical Spectral Devices) was applied to correct differences in the spectral drifts (at 1000 and 1800 nm) caused by inherent variation in detector sensitivities. Finally, reflectance data at 1-nm steps were obtained across the entire wavelength domain from 400 to 2500 nm. Five preprocessing methods were also tested based on their success in previous studies, namely FDR^54,68^, CR^69^, SNV^70^, MSC^71^, and DT^53^.

### Nitrogen and chlorophyll measurement

Total N was measured by dry combustion using an NC analyzer (Vario MAX cube, Elementar, Hanau, Germany). Aspartic acid was used as a standard in total N analysis.

Chlorophylls a and b were extracted from finely ground powder (5 mg) of freeze-dried leaf samples using *N*,*N′*-dimethylformamide (5 mL). After incubation for 24 h at 4 °C under dark conditions to allow complete decolorization, the samples were centrifuged at 2000×*g* for 30 min, and the absorbance of the supernatant was measured at 663.8 and 646.8 nm using a spectrophotometer (UV-1900, Shimadzu, Kyoto, Japan). Chlorophyll a and b contents were calculated using the equation of Porra et al. (1989)^72^.

### Regression models by machine learning algorithms

The flow of regression model generation was conducted as described by Sonobe et al. (2020)^54^, with a slight modification. For modelling, all measurements were divided into three groups, a training dataset (50%), a validation dataset (25%), and a test data dataset (25%), using a stratified sampling approach^73^. To ensure robust results, this approach was repeated 100 times before preprocessing the original reflectance and generating regression models based on machine learning algorithms.

When applying machine learning algorithms, a genetic algorithm (GA)-based approach^74^ is applied to select wavelengths using R ver. 3.6.3. that are effective for removing noninformative variables to obtain better and simpler prediction models. Regression models were then created from the selected bands using the following five methods: RF, SVM, Cubist, SGB, and KELM. To optimize the hyperparameters of these machine learning algorithms, Bayesian optimization was applied with the Gaussian process^75,76^ using R package “rBayesianOptimization” ver. 1.1.0. The information about the hyperparameters of these machine learning algorithms were shown in Supplementary Table S2.

RF was performed and optimized with R package “randomForestSRC” ver. 2.9.3 using the following five hyperparameters: number of trees; number of variables used to split nodes; minimum number of unique cases in a terminal node; maximum depth to which a tree should be grown; and number of random splittings. SVM was performed with the Gaussian radial basis function kernel and optimized with R package “e1071” using the following two hyperparameters: Regularization parameter (C) and kernel bandwidth (s). These were considered as user-defined hyperparameters using the R package “e1071” ver. 1.5-8. Cubist was performed and optimized with R package “Cubist” ver. 0.2.3 using the following two hyperparameters: Number of committee models (boosting iterations); number of neighbors. SGB was performed and optimized with R package “gbm” ver. 2.1.5 using the following four hyperparameters: Number of iterations and number of basis functions in the additive expansion; maximum depth of each tree; learning rate; and minimum number of observations in the terminal nodes of the trees. KELM was performed and optimized with MATLAB and Statistics Toolbox Release 2016a (The MathWorks, Inc., Natick, MA, USA; source code downloaded from https://www.ntu.edu.sg/home/egbhuang/) using the following two hyperparameters: Regulation coefficient (Cr) and kernel parameter (Kp).

The estimation accuracy of each method was evaluated based on the coefficient of determination (R^2^), root-mean-square error (RMSE), and ratio of performance to deviation (RPD). The quality of the prediction model was interpreted according to three classes of RPD^77–79^, as follows: RPD > 2 represents accurate prediction by the model, RPD of 1.4–2 represents fairly acceptable prediction, and RPD < 1.4 represent poor performance by the prediction model.

### Sensitivity analysis

Data-based sensitivity analysis (DSA) is proposed as a visualization approach for extracting human-understandable knowledge from supervised learning black box data mining models^80,81^. A black box analysis of the fitted models was performed with their machine learning algorithms by querying the fitted models with sensitivity samples and recording their responses, as previously described by Sonobe et al. (2020)^54^.

## Supplementary information

Supplementary Tables.

Supplementary Figures.
