# Supplementary material for: Dissection of hyperspectral reflectance to estimate nitrogen and chlorophyll contents in tea leaves based on machine learning algorithms
Source: Sci Rep. 2020 Oct 15;10:17360. doi: 10.1038/s41598-020-73745-2 (PMC7566634; doi:10.1038/s41598-020-73745-2)
Supplement: Supplementary file 2 — Supplementary Figures. [file 41598_2020_73745_MOESM2_ESM.pdf]

Supplementary Information

Dissection of hyperspectral reflectance to estimate nitrogen and chlorophyll contents in tea leaves based on machine learning algorithms

Hiroto Yamashita[1,2], Rei Sonobe[1*], Yuhei Hirono[3], Akio Morita[1] and Takashi Ikka[1*]

Correspondence to:
Rei Sonobe
sonobe.rei@shizuoka.ac.jp
Takashi Ikka
ikka.takashi@shizuoka.ac.jp

This PDF file includes:

Supplementary Figure S1 Typical pre-processing reflectance of green and albino yellow leaves with different nitrogen (N) statuses.

Supplementary Figure S2 Coefficient of determination (R^2) and root mean square error (RMSE) for each regression model and pre-processing of reflectance in nitrogen content.

Supplementary Figure S3 Coefficient of determination (R^2) and root mean square error (RMSE) for each regression model and pre-processing of reflectance in chlorophyll content.

Supplementary Figure S4 Data distribution of nitrogen (N) and chlorophyll (Chl) contents in tea mature leaves.

Supplementary Figure S5 Model performance and robustness for each pre-processing of reflectance in mature leaves.

Supplementary Figure S6 Detection of important hyper-parameter regions as model variables by data-based sensitive analysis (DSA) of each pre-processing in mature leaves.

Supplementary Figure S7 Correlation plots between leaf water and N contents in the dataset of this study.

Supplementary Figure S8 Detection of important hyperspectral parameter regions as model variables by data-based sensitive analysis (DSA).

Supplementary Figure S9 Photographs of “Koganemidori”, a cultivar with albino yellow leaves.

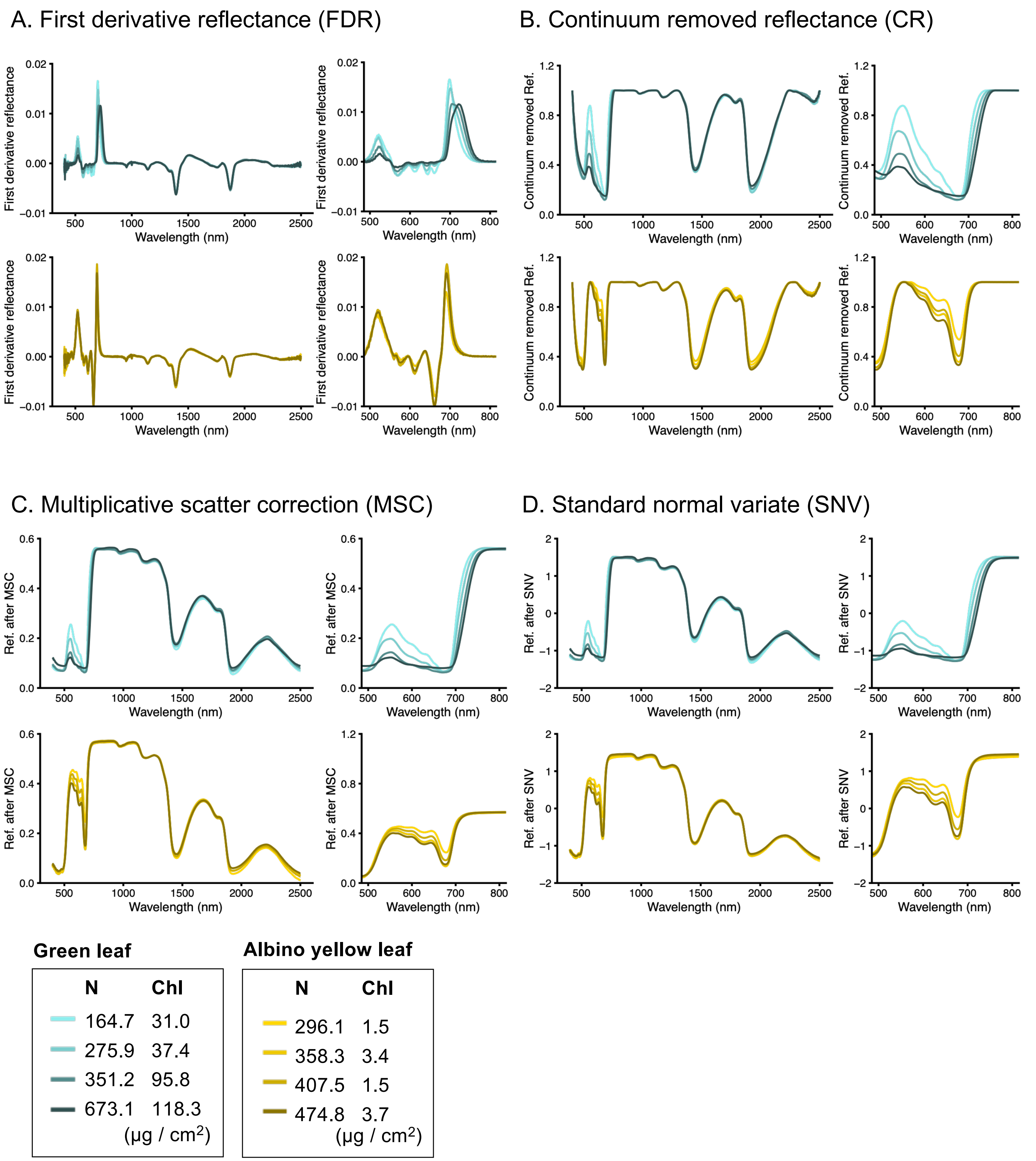

Supplementary Figure S1 Typical pre-processing reflectance of green and albino yellow leaves with different nitrogen (N) statuses.

N and chlorophyll contents in each spectrum are shown with different colors below the figure. Each figure consists of all measured wavelengths (left) and green peak and red edge ranges (right).

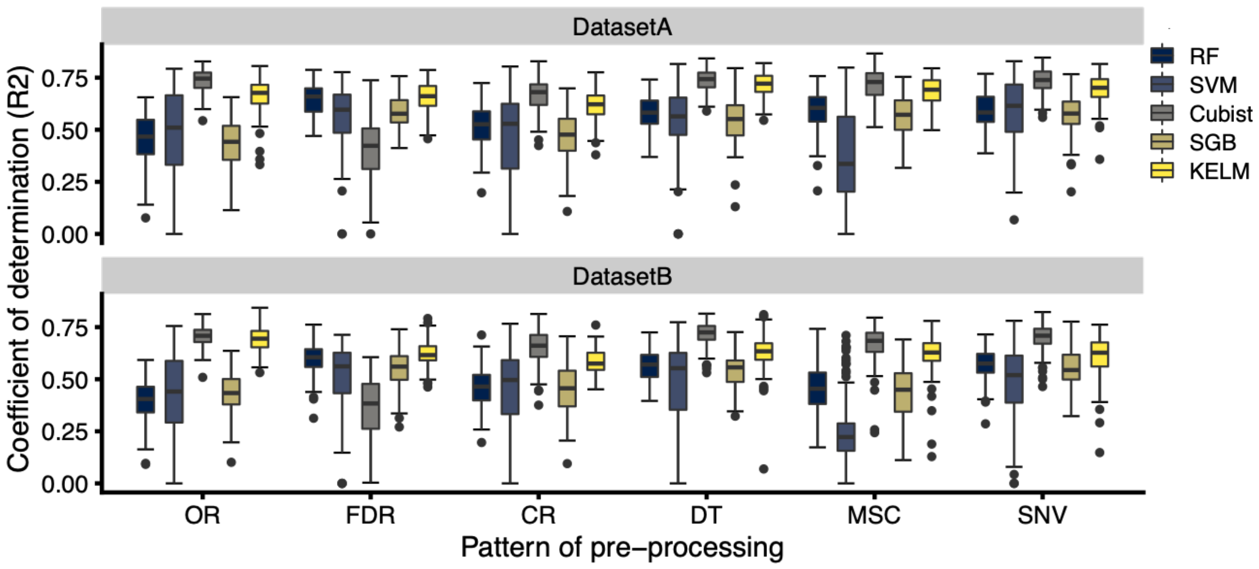

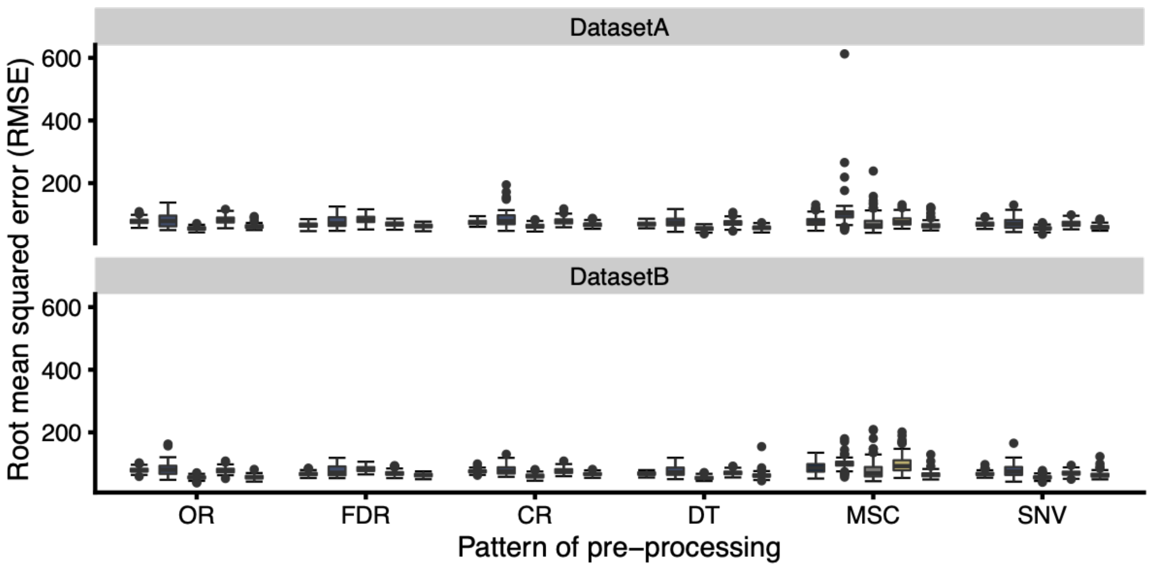

Supplementary Figure S2 Coefficient of determination (R^2) and root mean squared error (RMSE) for each regression model and pre-processing of reflectance in nitrogen content.

Figures are plots of the R^2 and RMSE values in each repeat.

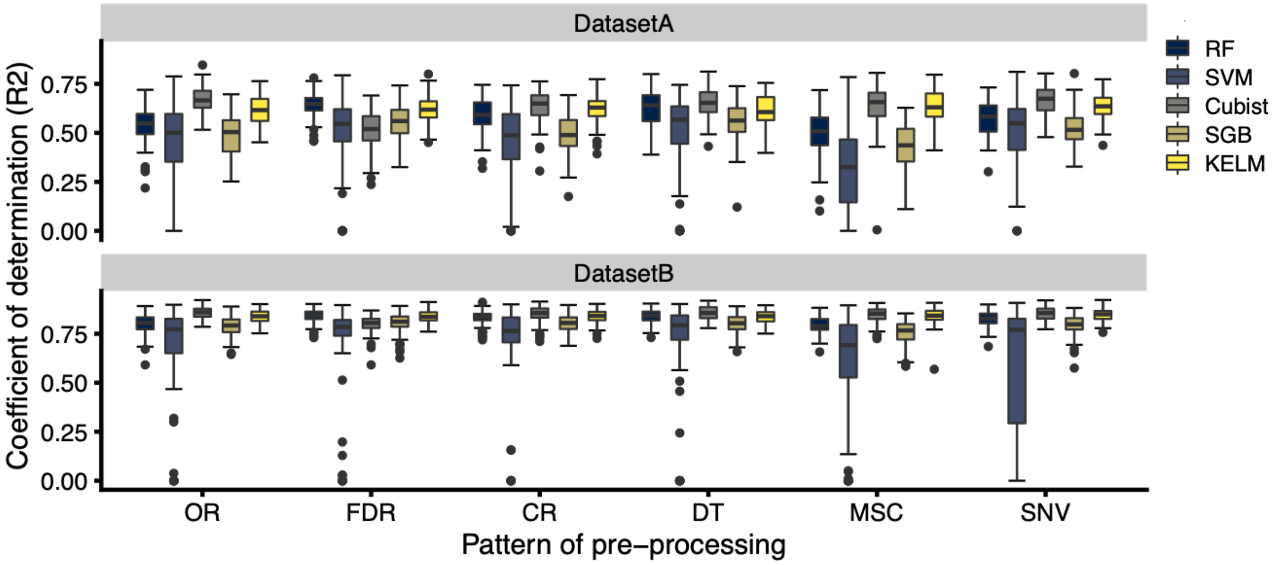

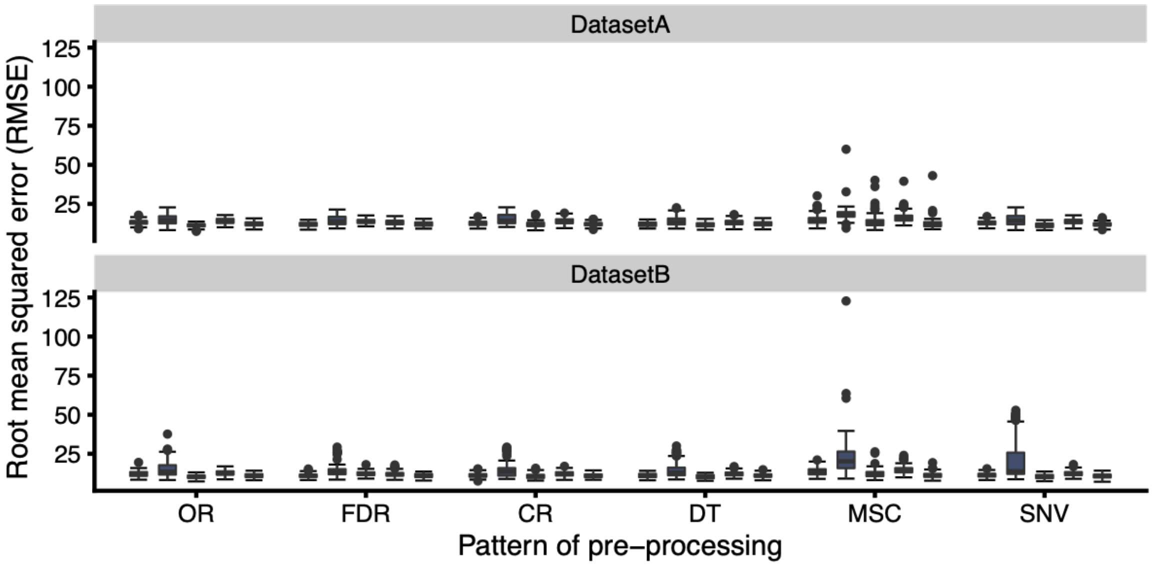

Supplementary Figure S3 Coefficient of determination (R^2) and root mean squared error (RMSE) for each regression model and pre-processing of reflectance in chlorophyll content.

Figures are plots of the R^2 and RMSE values in each repeat.

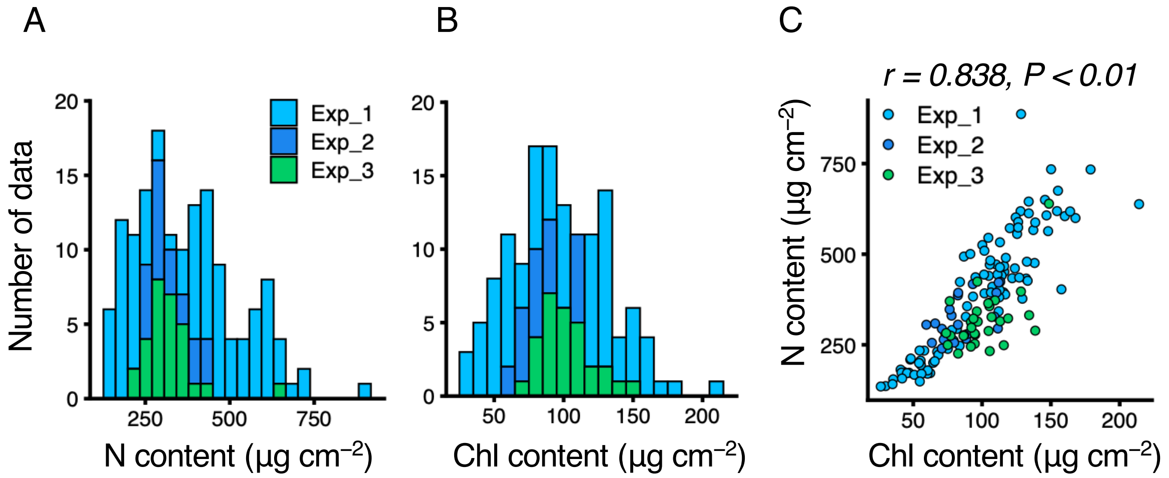

Supplementary Figure S4 Data distribution of nitrogen (N) and chlorophyll (Chl) contents in tea mature leaves.

Histgram of (A) N and (B) Chl contents in all experiments. (C) Correlation plots between N and Chl content.

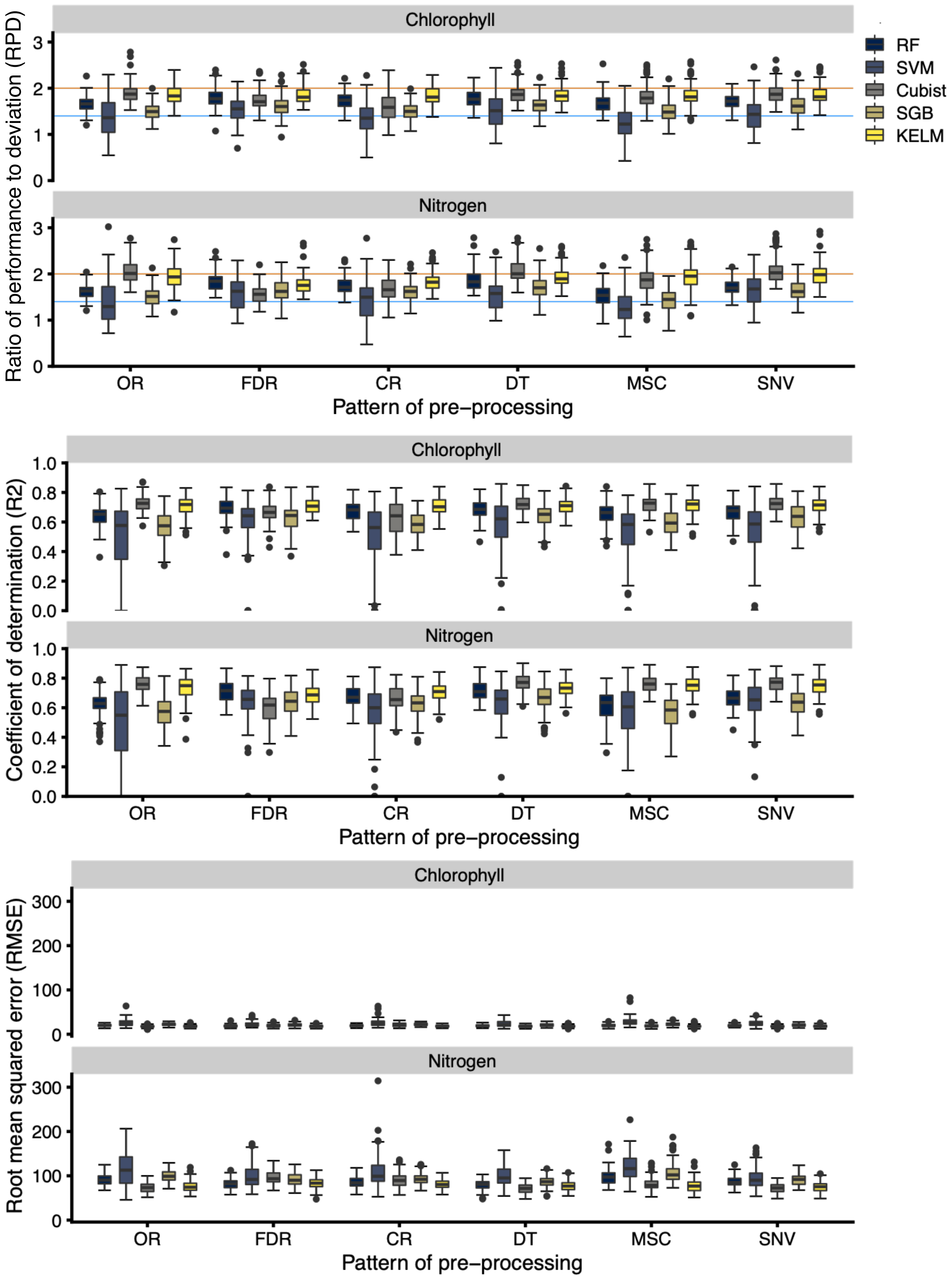

Supplementary Figure S5 Model performance and robustness for each pre-processing of reflectance in mature leaves.

Modelling with nitrogen and chlorophyll contents as objective variables was performed using explanatory variables. The ratio of performance to deviation (RPD), coefficient of determination (R^2), and root mean squared error (RMSE) were applied to evaluate the accuracy of each model. A stratified sampling approach for modeling was repeated 100 times to obtain robust results. Figures are plots of the RPD, R2, and RMSE values in each repeat. Orange and blue line indicates RPD values of 1.4 and 2.0, respectively, as accuracy thresholds.

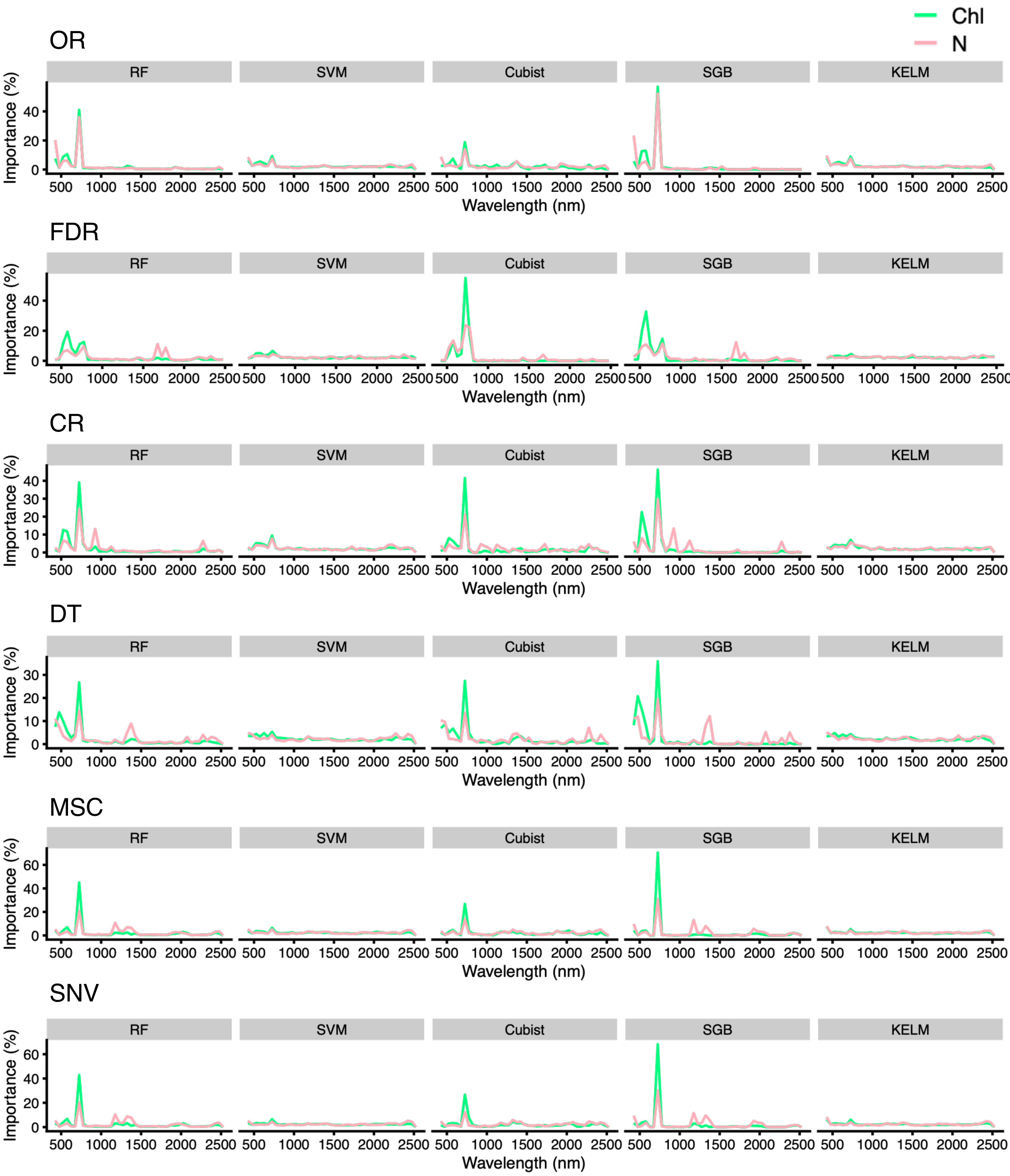

Supplementary Figure S6 Detection of important hyper-parameter regions as model variables by data-based sensitive analysis (DSA) of each pro-processing in mature leaves.

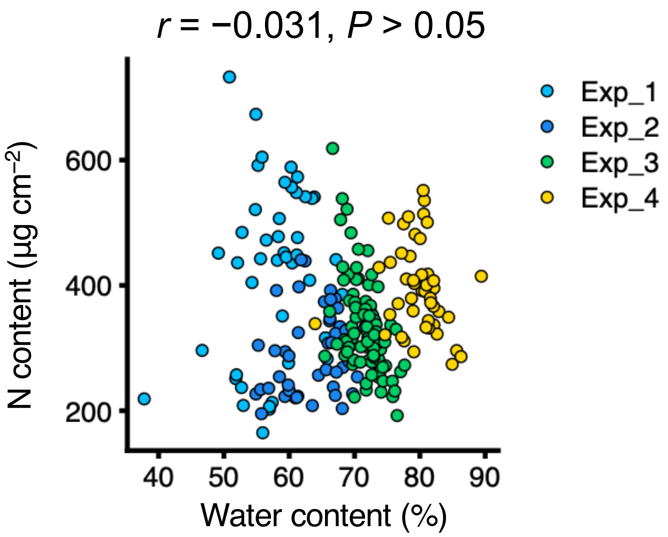

Supplementary Figure S7 Correlation plots between leaf water and N contents in the dataset of this study

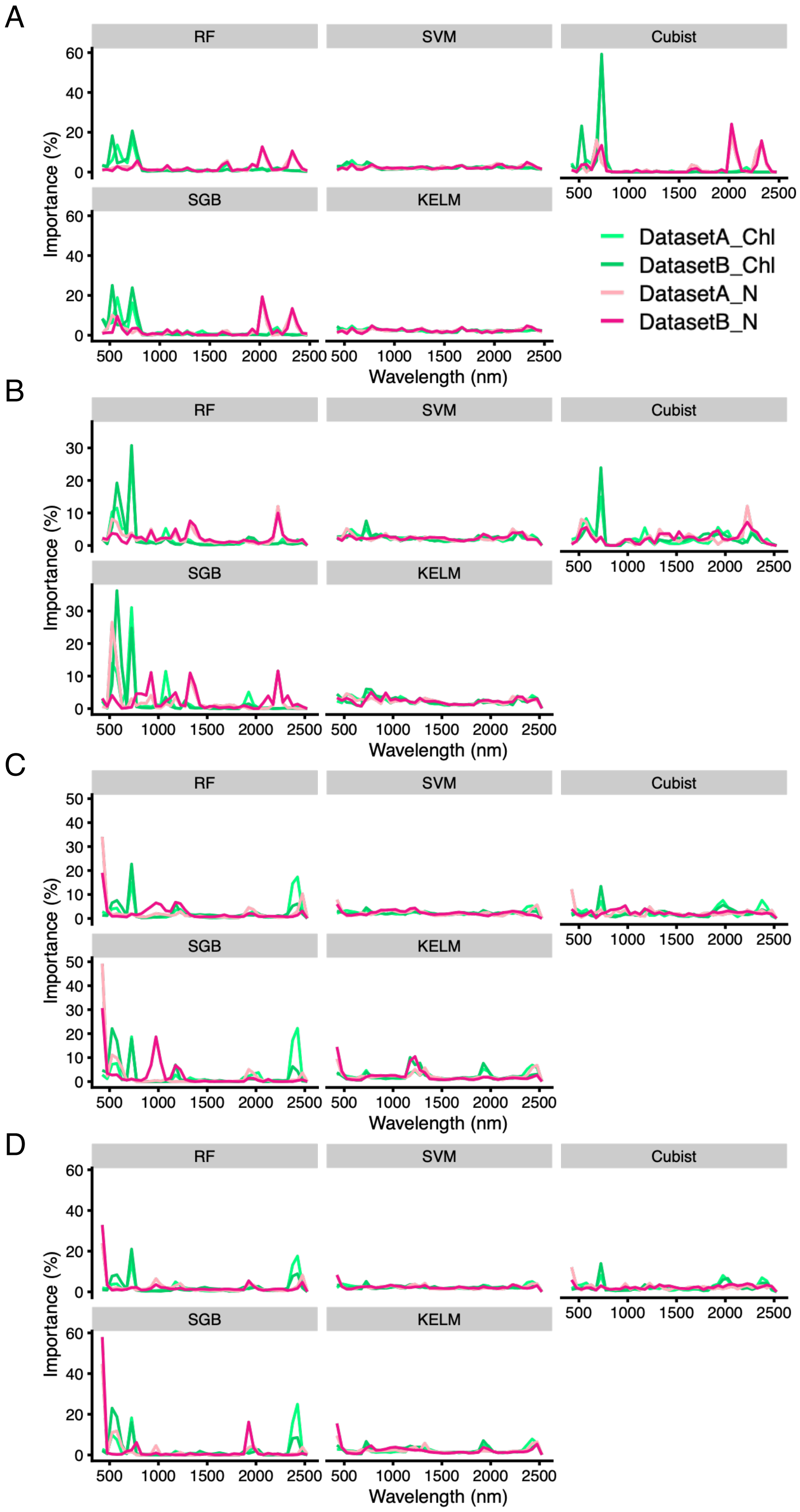

Supplementary Figure S8 Detection of important hyperspectral parameter regions as model variables by data-based sensitive analysis (DSA).
(DSA results for (A) first derivative spectra (FDR), (B) continuum removed reflectance (CR), (C) multiplicative scatter correction (MSC), and (D) standard normal variate (SNV) were visualized as explanatory variable in each model.

A B

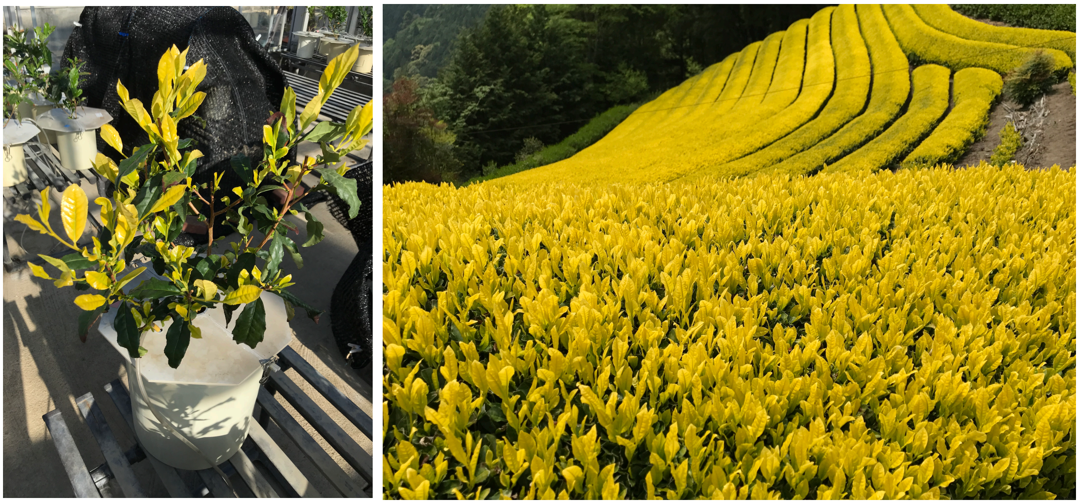

Supplementary Figure S9 Photographs of “Koganemidori”, a cultivar with albino yellow leaves. (A) Individualy cutivated by hydroponics used in this study; (B) appearance at the tea garden level.
